# HELLP syndrome, intracerebral hemorrhage, and hemophagocytic syndrome after cesarean section in a pregnant patient with severe preeclampsia: a case report

**DOI:** 10.1186/s12884-023-05462-3

**Published:** 2023-02-28

**Authors:** Minghe Tan, Siqi Wang, Qingshu Li, Ruixue Yuan, Maoji Zhao, Jun Cao

**Affiliations:** 1 Department of Anesthesiology, The First Affiliated Hospital of Chongqing Medical University, Chongqing, 400016 China; 2 Department of Pathology, School of Basic Medicine, Chongqing Medical University, Chongqing, 400016 China

**Keywords:** HELLP syndrome, Intracerebral hemorrhage, Hemophagocytic syndrome, Severe preeclampsia

## Abstract

**Background:**

Pregnancy-related intracranial hemorrhage (ICH) is a rare but potentially life-threatening event with complex and varied cause, such as HELLP syndrome and hemophagocytic syndrome.

**Case presentation:**

A 33-year-old patient underwent a cesarean section with a preliminary diagnosis of "severe preeclampsia and class3 HELLP syndrome ". The patient had poor response to language before surgery, and the catheter drainage fluid was hematuria. Later, the surgeon reported severe bleeding in the operation. Following thromboelastography (TEG) result and postoperative laboratory tests confirmed class1 HELLP syndrome and ICH occurred on the second day after the surgery, and hemophagocytic syndrome was diagnosed during subsequent treatments.

**Conclusion:**

For patients with HELLP syndrome, we should pay attention to their coagulation condition. The coagulation tests and platelet counts should be repeated if their clinical presentation changed. Those with neurological alarm signs should receive CT or MRI scan. If a pregnant woman had prolonged hemocytopenia and thrombocytopenia, not only the HELLP but also the hemophagocytic syndrome should be considered.

## Background

Pregnancy-related intracranial hemorrhage (ICH) is a rare but potentially fatal disease in pregnant women [1, 2]. Compared with other complications during pregnancy, the prognosis of pregnancy-related ICH in both mothers and fetus are worse [1, 2]. HELLP syndrome is characterized by hemolysis, elevated liver enzyme levels and low platelet count (PLT), maybe significantly associated with poor outcomes in pregnancy-associated ICH patients [2, 3]. Hemophagocytic syndrome is characterized by fever, hepatosplenomegaly, cytopenia and activated macrophages in haemopoietic organs. The most cases are in children, cases in adults or during pregnancy and childbirth are rare [4]. The simultaneous occurrence of hemophagocytic syndrome, HELLP syndrome and ICH had not been reported before, we have encountered such a case, now report as follows.

## Case presentation

A 32-year-old patient was admitted to the emergency department due to G_1_P_0_ threatened labor at 39 weeks with severe preeclampsia. Her medical and family history was unremarkable, no hypertension or ICH history. Physical examination on admission: T( body temperature) 36.7 ℃, P(pulse rate) 102 times min^−1^, R(respiratory rate) 20 times min^−1^, BP(blood pressure) 188/109 mmHg. The patient was treated with 20% mannitol 250 ml, 5% magnesium sulfate 100 ml, and urapidil 50 mg to prevent eclampsia. Her blood pressure was controlled between 140–160/100–110 mmHg and the headache relieved. Relevant examinations were completed immediately, blood routine showed mild thrombocytopenia(PLT 132 × 10^9^ L^−1^), routine biochem examination indicated suspicious hemolysis[ lactate dehydrogenase (LDH) 2136U L^−1^, broken erythrocytes were not found] and elevated liver enzymes [alanine aminotransferase (ALT)186U L^−1^, aspartate aminotransferase (AST) 372U L^−1^]. Urinalysis indicated hematuria and proteinuria. Severe preeclampsia and class3 HELLP syndrome (the mildest type) were diagnosed. Considering that the fetus was full-term but unable to deliver vaginally within a short time, the patient was intended to terminate the pregnancy by cesarean section under epidural anesthesia. However, the patient had poor response to language when entered the operating room 5 h after admission, the GCS score is 13 points, her verbal response and eye-opening response each lose 1 point, and the catheter drainage fluid was hematuria. The anesthesiologist decided to perform general anesthesia after comprehensive evaluation. Before anesthesia induction, arterial blood was collected for thromboelastography (TEG) detection. The patient underwent a cesarean section and a girl was delivered. During the operation, the surgeon reported severe bleeding from the surgical incision. The TEG result received after surgery showed low PLT or function, low fibrinogen level or function and coagulation disorder (R 8.4 min, K 5.3 min, Angel 39.2°, MA 43.6 mm, CI-0.7, G3.9 k D/SC). Combined TEG result with significant increased liver enzymes before surgery, we suspected serious HELLP syndrome was the reason of bleeding tendency [5]. The patient was transported to the central ICU with tracheal tube after surgery.

The laboratory test results in ICU indicated hemolysis (H), elevated liver enzymes (EL), low PLT (LP) (PLT 30 × 10^9^ L^−1^) and coagulation disorder, the class1 HELLP syndrome (the most severe form) was verified. Blood transfusion (5U suspended red blood cells and 1U platelets) was adopted. The patient's PLT increased, liver enzymes decreased, coagulation function returned to normal except D-dimers and fibrinogen degradation Products (FDP) (PLT 70 × 10^9^ L^−1^, ALT 90U L^−1^, AST 139U L^−1^, LDH 1753U L^−1^, FDP 15.5ug ml^−1^, D-dimers 4.65 mg L^−1^), endotracheal tube was removed 1 day later.

**Fig. 1:**
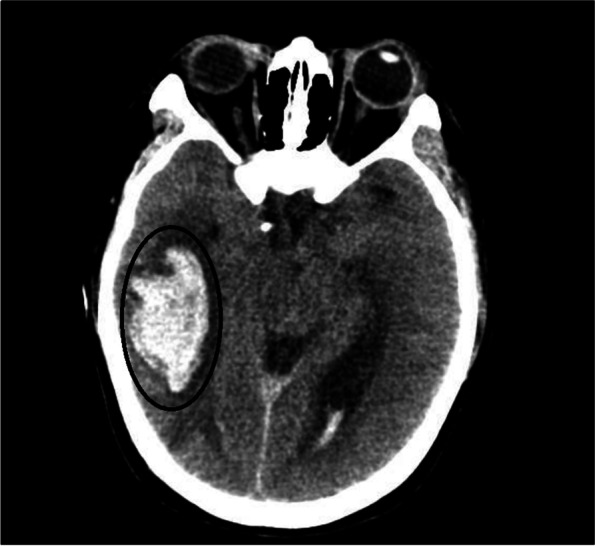
CT scan of cerebral hemorrhage

The laboratory test results improved (PLT 74 × 10^9^ L^−1^, FDP15.0ug ml^−1^, D-dimers4.82 mg L^−1^, others are normal) 2 days after surgery. The blood pressure was well controlled with continuous urapidil pumping. However, the patient had repeated attacks of headache and convulsions, each time lasting for tens of seconds, and the Babinski's sign, Buchner's sign, and Kirschner's sign were positive. Considering the possibility of eclampsia, magnesium sulfate, urapidil and other symptomatic treatments were given. Then skull CT suggests ICH in the right temporal lobe and basal ganglia (Fig. 1). The CTA and CTV showed no obvious cerebral vascular malformation. Then she was admitted to the operation room for clearance of intracerebral hematoma in emergency. The patient did not complain obvious headache after surgery and vital signs were stable after surgery. She was transferred out of ICU 6 days later.

**Fig. 2:**
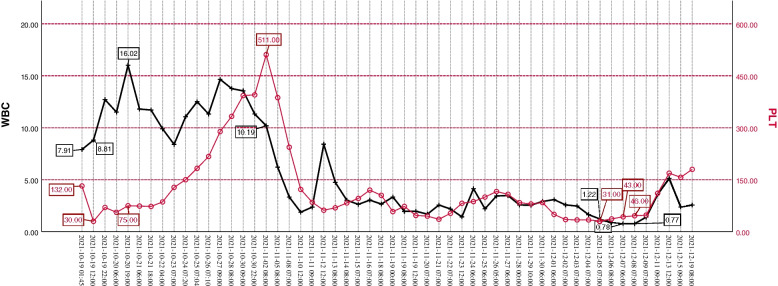
WBC and PLT trend chart

**Fig. 3:**
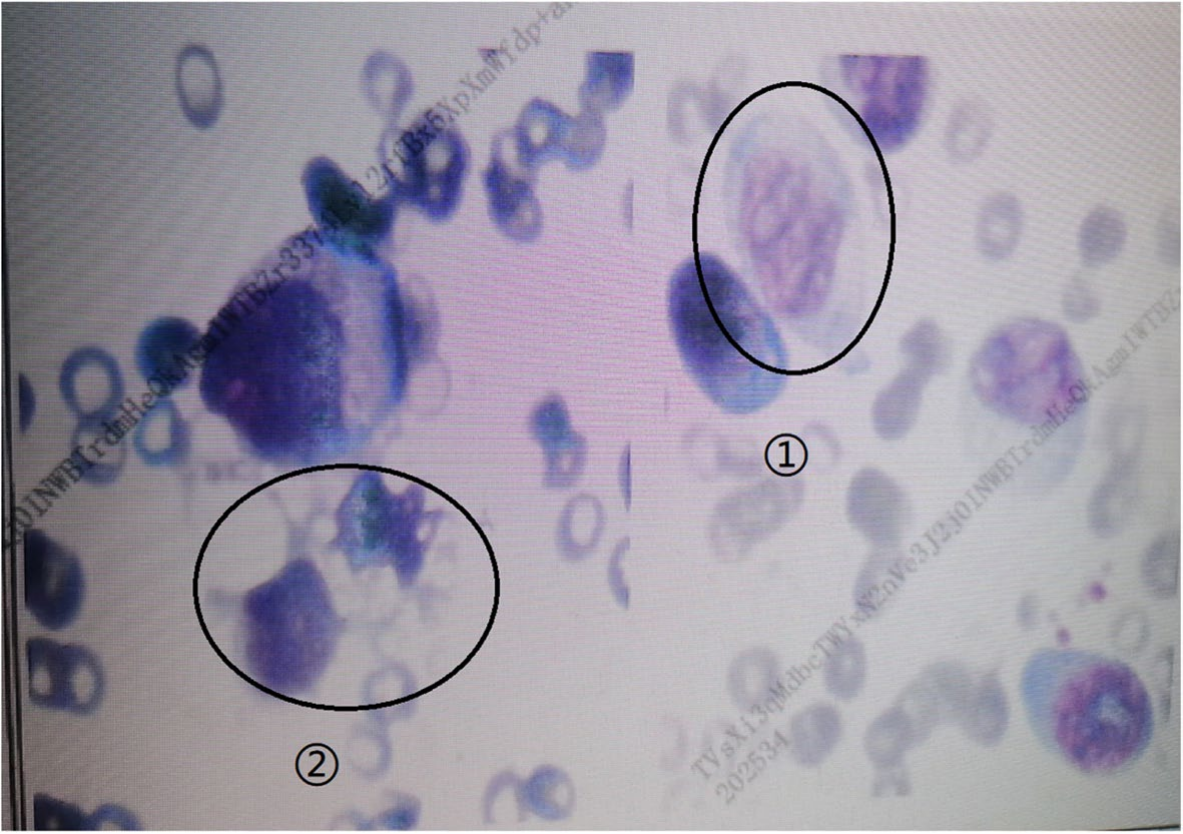
bone marrow biopsy (①Activated monocytes; ②Tissue phagocytes)

About 14 days after admission, the patient developed a progressive decline in blood cells and PLT (Fig. 2), and developed suspected septic shock(somnolence, T 39.7℃, BP 81–99/35-68 mmHg, HR 172–180 times min^−1^, R 46 times min^−1^, procalcitonin: 34.13 ng ml ^−1^) and coagulation disorder [Prothrombin time (PT) 20.4 s, International Normalized Ratio (INR) 1.76, activated partial thromboplastin time (APTT) 67.3 s, FDP 237.8 μg ml^−1^, D-dimers: 93.62 mg L^−1^]. The aggressive platelet transfusion and the use of colony stimulating factor had been adopted, ceftazidime, meropenem, and vancomycin were used to treat the infection of the patients. At that time, we considered it was caused by HELLP syndrome or infection-related hemophagocytic syndrome. But the auxiliary examination did not meet the diagnostic criteria of hemophagocytic syndrome (abdominal CT and bone marrow biopsy were normal, repeatedly searches for viral and bacterial infectivity indicators were failed). Prophylactically, steroid pulse therapy and intravenous immunoglobulins therapy were administered. We complete ferritin, interleukin 2 receptor (IL-2R), NK cell activity and genetic mutation associated with hemophagocytic syndrome. After 49 days in hospital, hemophagocytic syndrome was diagnosed [persistent fever (> 38·5 °C); severe pancytopenia; hypertriglyceridemia; low and absent NK cell; ferritin concentrations on the 34th day:16,279.0 ng mL^−1^, IL-2R concentrations on the 34th day: 6757.0 IU ml^−1^; abdominal CT on the 41th day showed splenomegaly; bone marrow biopsy on the 42th day showed hemophagocytosis (Fig. 3)] [6, 7]. However, there were no abnormal genes related to hemophagocytic syndrome, no tumor markers in the blood system been found. The EBV (Epstein barr virus)-DNA, CMV (cytomegalo virus)-DNA and other viruses and bacterial infection indicators were negative. The ANCA (Anti Neutrophellol Cytoplasmic Antibody), antinuclear antibody spectrum and humoral immunity were normal. So, the cause of hemophagocytic syndrome is still unknown. The patient was discharged in 2 months after admission finally.

## Discussion and conclusion

The patient underwent a general anesthesia caesarean section to terminate the pregnancy because of severe preeclampsia and class3 HELLP syndrome. Her platelet count and coagulation function were in the lower limit of normal range on admission. But the preoperative TEG results and postoperative blood routine indicated a sharp decrease coagulation function within 5 h. The PLT decreased form 132 × 10^9^ L^−1^ to 30 × 10^9^ L^−1^, with a high incidence of serious complications [8]. Then class 1HELLP syndrome was diagnosed. The patient was treated with aggressive blood product transfusion after surgery. Although the patient's condition improved, suspicious eclampsia attack occurred 2 days after surgery and eventually confirmed as ICH, as the patient had a poor response to language before cesarean section, the ICH may occur earlier in a context of severe hypertension and HELLP syndrome. And then hemophagocytic syndrome gradually appeared, but the cause is still unknown.

The diagnosis of HELLP syndrome mainly depends on laboratory tests.

There are two major definitions for diagnosing the HELLP syndrome. In the Tennessee Classification System, Sibai strictly requires the diagnostic criteria for HELLP syndrome to be platelet count < 100 × 10^9^/L, AST ≥ 70 UI/L and LDH ≥ 600 UI/L. In the Mississippi-Triple Class System, it requires the presence of thrombocytopenia (perinatal platelet nadir ≤ 150,000 cells/µL), evidence of hepatic dysfunction (ALT ≥ 40 IU/L, AST ≥ 40 IU/L, or both, with LDH ≥ 600 IU/L), and evidence of hemolysis (increased LDH level, progressive anemia).There are a more detailed classification based on the nadir of platelets count any time during the course of the disease in the Mississippi-Triple Class System, class 1 requires platelet count ≤ 50 × 10^9^/L, class 2 requires platelet count between50 × 10^9^/L and 100 × 10^9^/L and class 3 requires platelet count ≥ 100 × 10^9^/L. Besides, class 1 and class 2 require LDH > 600 U/L and AST ≥ 70 U/L. It should be noted that elevated LDH is a hint of hemolysis [9–11]. However, the quickly decline of PLT and coagulation function suggested that judging patient's condition only by laboratory results is not reliable in such cases. In addition, when above sharply changes accompany with the elevated LDH and liver enzyme levels, bleeding tendency, severe preeclampsia or eclampsia, the HELLP syndrome should be considered even PLT and coagulation test are normal [9, 10].

Pregnancy-related hemophagocytic syndrome is a very rare disease. According to Liu et al. [4], only 81 pregnancy-related hemophagocytic syndrome cases have been reported until 2021. There is no consensus on the diagnosis and treatment of hemophagocytic syndrome in pregnant patients at present. The clinical manifestations are almost similar to HELLP syndrome, so it is often misdiagnosed, especially when HELLP syndrome co-existed [4]. The cause of the disease is unclear, genetic factors or infection as trigger factors are accepted by most people. In adults, mostly hemophagocytic syndromes are secondary to EBV and CMV infection, the pregnancy may be a contributor. Pregnancy itself is a systemic inflammatory response, and preeclampsia is considered a systemic inflammatory disease. Inflammatory reactions and cytokine storm may induce or exacerbate hemophagocytic syndrome.In contrast to non-pregnant cases, the incidence of pregnancy-related hemophagocytic syndrome is very low and 37% of pregnancy-related hemophagocytic syndrome are unexplained, and it has a high mortality rate, so when a pregnant woman presents with prolonged hemocytopenia and thrombocytopenia, not only the HELLP syndrome but also the hemophagocytic syndrome should be considered [4, 6].

Because the PLT may decline very quickly on HELLP syndrome, we should also pay attention to the patient's clinical manifestations to evaluate the patient's coagulation condition and repeat the laboratory tests [5, 12]. If the patients had bleeding possibilities such as mental state changes, gingival bleeding and hematuria, TEG examination is a good choice. The anesthetic management of these patients is complex, and the risks and benefits of each anesthetic technique must be considered based on a full understanding of the pathophysiological conditions of these patients. Platelet counts higher than 80,000 per cubic millimeter indicate that spinal and epidural anesthesia is safe in the absence of risk factors (anticoagulants, antiplatelet agents, abnormalities in coagulation or platelet function, or rapid platelet decline), these patients should be closely monitored after spinal puncture, as their coagulation function may change dramatically. General anesthesia should be selected if there is an immediate threat for the mother and the fetus, such as eclampsia, pulmonary edema, or an altered level of consciousness, or for contraindications to regional anesthesia, such as coagulation disorder. In any case, frequent neurological evaluation is necessary until the coagulopathy is resolved, and those with neurological alarm signs should immediately consult a neurologist and explore the patient by CT or MRI. [5, 10, 12]. Patients with class 1 HELLP syndrome should keep PLT above 50 × 10^9^ L^−1^ to avoid the risk of bleeding or ICH [10, 13].Any type of ICH should be managed after diagnosis to prevent rebleeding or enlargement of the hematoma, including the use of vasoactive drugs to control blood pressure and prompt surgical treatment [14].

In summary, anesthesiologist should learn from the case that if a patient with HELLP syndrome had very rapid deterioration in coagulation function and serious bleeding incident, some rare possibilities should be considered, such as hemophagocytic syndrome. Although no definitely relationship has been reported, the case gave us the reason to suspect the link between HELLP syndrome and hemophagocytic syndrome. Perhaps HELLP syndrome is a trigger for hemophagocytic syndrome [4], but further evidence needs to be collected in the future.

## Data Availability

All data related to this report are available from the corresponding author on reasonable request.

## Abbreviations

ICH: Pregnancy-related intracranial hemorrhage
PLT: Platelet count
T: Body temperature
P: Pulse rate
R: Respiratory rate
BP: Blood pressure
LDH: Lactate dehydrogenase
ALT: Alanine aminotransferase
AST: Aspartate aminotransferase
FDP: Fibrinogen degradation Products
PT: Prothrombin time
INR: International normalized ratio
APTT: Activated partial thromboplastin time
IL-2R: Interleukin 2 receptor
EBV: Epstein barr virus
CMV: cytomegalo virus
ANCA: anti neutrophellol cytoplasmic antibody
